# Optimal stimulation settings for CMAP scan registrations

**DOI:** 10.1186/1749-7221-7-4

**Published:** 2012-06-18

**Authors:** Ellen M Maathuis, Robert D Henderson, Judith Drenthen, Nicole M Hutchinson, Jasper R Daube, Joleen H Blok, Gerhard H Visser

**Affiliations:** 1 Department of Clinical Neurophysiology, Erasmus MC, University Medical Center Rotterdam, P.O. Box 2040, 3000 CA, Rotterdam, The Netherlands; 2 Department of Neurology, Royal Brisbane & Women’s Hospital, Queensland, Australia; 3 Department of Neurology, Mayo Clinic, Rochester, MN, USA

**Keywords:** CMAP scan, Stimulus–response curve, Settings, Stimulus number, Stimulus frequency, Stimulus duration

## Abstract

**Background:**

The CMAP (Compound Muscle Action Potential) scan is a non-invasive electrodiagnostic tool, which provides a quick and visual assessment of motor unit potentials as electrophysiological components that together constitute the CMAP. The CMAP scan records the electrical activity of the muscle (CMAP) in response to transcutaneous stimulation of the motor nerve with gradual changes in stimulus intensity. Large MUs, including those that result from collateral reinnervation, appear in the CMAP scan as so-called steps, i.e., clearly visible jumps in CMAP amplitude. The CMAP scan also provides information on nerve excitability. This study aims to evaluate the influence of the stimulation protocol used on the CMAP scan and its quantification.

**Methods:**

The stimulus frequency (1, 2 and 3 Hz), duration (0.05, 0.1 and 0.3 ms), or number (300, 500 and 1000 stimuli) in CMAP scans of 23 subjects was systematically varied while the other two parameters were kept constant. Pain was measured by means of a visual analogue scale (VAS). Non-parametric paired tests were used to assess significant differences in excitability and step variables and VAS scores between the different stimulus parameter settings.

**Results:**

We found no effect of stimulus frequency on CMAP scan variables or VAS scores. Stimulus duration affected excitability variables significantly, with higher stimulus intensity values for shorter stimulus durations. Step variables showed a clear trend towards increasing values with decreasing stimulus number.

**Conclusions:**

A protocol delivering 500 stimuli at a frequency of 2 Hz with a 0.1 ms pulse duration optimized CMAP scan quantification with a minimum of subject discomfort, artefact and duration of the recording. CMAP scan variables were influenced by stimulus duration and number; hence, these need to be standardized in future studies.

## Background

**Figure 1:**
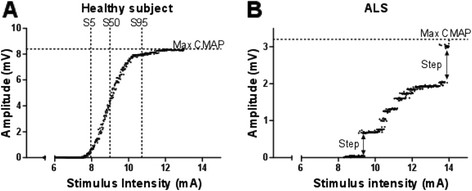
**A:** CMAP scan with 500 stimulus–response combinations collected in a normal subject. CMAP amplitude increases with increasing stimulus intensity. The maximum CMAP amplitude is indicated with the horizontal dotted line. S5, S50, and S95 are the stimulus intensities that elicited responses with a size of 5, 50, and 95 percent of the maximum CMAP, respectively (dotted vertical lines). **B:** CMAP scan in a 76 year old patient with amyotrophic lateral sclerosis, six months after diagnosis. The CMAP scan clearly differs from the CMAP scan of the healthy subject due to the many steps in the curve. The maximum CMAP amplitude is 3.2, as indicated with the horizontal dotted line. The two largest steps are indicated with arrows. The largest step in this CMAP scan is 0.9 mV which comprises 28% of the maximum CMAP. The step percentage is the summation of all steps in the CMAP scan. In this case the sum of all steps is almost 2.2 mV which is 68% of the maximum CMAP. The step percentage of this CMAP scan is therefore 68%.

The CMAP (Compound Muscle Action Potential) scan is a non-invasive neurophysiological tool, which records the electrical activity of a muscle in response to repetitive transcutaneous stimulation of the motor nerve [1,2]. It is based on the fact that motor units (MUs) differ with respect to the stimulus intensity that is required to activate them, i.e., they have differing thresholds. If the stimulus intensity is gradually increased from subthreshold to supramaximal values, all MUs in the muscle are successively activated. Plotting the size of the resulting CMAP against the stimulus intensity normally results in a smooth, sigmoid curve: the CMAP scan (Figure 1A).

Large MUs, including those that result from collateral reinnervation, will show in the CMAP scan as so-called steps, i.e., clearly visible jumps in CMAP amplitude (Figure 1B). Henderson et al. showed that patients with amyotrophic lateral sclerosis (ALS) had more and larger steps in the CMAP scan than healthy controls [2]. Using a Bayesian algorithm, an estimation of the number of functional MUs in a muscle (motor unit number estimation, MUNE) can be derived from the CMAP scan [3]. MUNE is particularly useful for the monitoring of neurogenic disorders and traumatic lesions, which are characterized by a loss of functioning MUs. The CMAP scan also provides basic information on nerve excitability that cannot be obtained with standard nerve conduction studies. With increasing SIs the recorded CMAP will increase, depending on the excitability of individual motor units. The excitability parameters of the CMAP scan are the stimulus intensities that elicit 5%, 50% and 95% of the maximum CMAP (S5, S50 and S95 respectively, see Figure 1A) and the range between the S5 and S95.

This information can contribute to the diagnosis and monitoring of demyelinating diseases such as Guillain-Barré syndrome (GBS) [1]. Because all MUs contribute, the CMAP scan is not affected by sample bias that would favor certain subpopulations of MUs over others.

A lot of research on the effect of stimulus duration on nerve excitability has been done and it is well known that using a shorter stimulus duration results in a higher stimulus intensity needed to elicit the same CMAP amplitude [4-6]. Little is known about the influence of stimulus parameters such as stimulus frequency and the total number of stimuli on the properties of trains of recorded CMAPs, and, hence, on the CMAP scan. Especially the effect on the amount and size of steps in the CMAP scan is not known. To develop the CMAP scan as a clinical tool, the effect of the stimulus settings on the CMAP scan and its quantification must be defined to enable standardized collection of normal values and comparison of CMAP scan data between medical centers. The aim of the present study was to determine the optimal stimulus parameters settings for frequency, pulse duration and number, balancing between minimal subject discomfort, artefacts, and recording duration, while optimizing standardized quantification of the CMAP scan.

## Methods

### Subjects and design

The study was performed in Rotterdam, the Netherlands, and Brisbane, Australia. In total, 23 subjects were included (age, 25–67 years). In 17 of these (11 in Rotterdam, 6 in Brisbane), the median nerve was studied and in the other 6 (all Brisbane), ulnar nerve recordings were made. The effect of stimulus frequency (1, 2, and 3 Hz) and stimulus duration (0.05, 0.1, and 0.3 ms) was studied in Rotterdam. The effect of the number of stimuli (300, 500, and 1000) was studied in Brisbane. For each parameter, the three values were chosen such that they represented both realistic extremes and the expected optimum. For example, based on our previous work, we already knew that using fewer that 300 stimuli often results in spurious steps, while using more than 1000 makes the duration of the registration impractically long for diagnostic purposes.

Carpal tunnel syndrome (for median nerve testing) and ulnar neuropathy (for ulnar nerve testing) were excluded by means of conventional nerve conduction studies. None of the subjects had symptoms or signs of neurological disease. The experimental protocol was approved by the institutional Medical Ethics Committees in Rotterdam and Brisbane. All subjects gave informed consent.

### Recordings

The CMAP scan was recorded using a dedicated program on a Nicolet VikingSelect EMG system (CareFusion, San Diego, CA). This program allows parameters such as stimulus frequency, duration, number, and recording direction (upwards or downwards) to be changed easily. It displays and stores the absolute area and amplitude of the negative peak of the elicited CMAPs and the relative area and amplitude (as percentage of the area or amplitude of the recorded maximal CMAP), of each of the responses.

CMAP scan recordings were obtained from the thenar muscles for the median nerve and the hypothenar muscles for the ulnar nerve using 10 mm diameter, silver-silver chloride cup electrodes. The active electrode was placed over the muscle belly, and optimized for maximum CMAP size, negative onset, and biphasic shape. The reference electrode was placed on the metacarpal-phalangeal joint of the thumb for the median nerve and on the proximal interphalangeal joint of the fifth digit for the ulnar nerve. The ground electrode (self-adhesive surface electrode) was placed on the dorsum of the hand. In Rotterdam, the stimulator consisted of two 6 x 20 mm rectangular felt electrodes with an interelectrode distance of 20 mm, attached with a strap to the wrist with a minimum of surface compression. In Brisbane, the stimulating electrodes were 10 mm, silver-silver chloride cup electrodes taped to the skin. In both centers, the stimulator was applied to the wrist at the point of lowest threshold, an important factor to ensure reliable and reproducible quantification of stimulus intensity variables since stimulus intensity variables depend strongly on the relative location of the stimulus electrodes to the axons in the nerve trunk. The hand was immobilized and the thumb or fifth finger was taped to the side of the hand to avoid movement artefacts. Subjects were asked to remain relaxed, silent, and motionless during the recordings.

Recordings started with the determination of the lowest stimulus intensity (S0), measured in mA, that elicited an all-or-none response of the lowest-threshold MU. Next, the maximum CMAP was determined by increasing the stimulus intensity until the recorded CMAP was maximal and then turned up by another 30% to ascertain that all MUs had been activated (supramaximal stimulation). Third, the lowest stimulus intensity (S100 or maximal stimulation) at which the maximum CMAP could be recorded was determined and rounded to the next-highest integer value in mA. The final preparatory step consisted of a test scan of 30 stimuli to confirm that S0 and S100 were set correctly. If necessary, S0 and S100 were adjusted to ensure that the scan covered the entire CMAP range.

The experimental testing comprised a series of three CMAP scans in which one of the stimulus parameters was systematically varied. Each session started with standard settings (2 Hz, 1 ms, 500 stimuli), followed by the remaining two variants of the parameter under study. When one parameter was varied, all others were kept at the standard settings. The low and high settings of each parameter were studied alternately in subsequent subjects to avoid systematic bias due to fatigue or other order-dependent effects.

There was a pause of at least 3 minutes between recordings. After each CMAP scan, the eleven subjects studied in Rotterdam indicated the severity of experienced pain on a visual analogue scale (VAS) [7], which ranged from 0 (no pain) to 10 (most severe pain conceivable).

The CMAP scans performed with the standard settings were used for the evaluation of intercenter variability.

### Data analysis

All CMAP scans were analysed in Rotterdam. A quality check on the data was performed in Excel 2003 (Microsoft, Redmond, WA, USA) to identify obvious movement artefacts that were then removed by deleting the affected samples from the CMAP scan. For quantitative analysis, the data were subsequently imported in MATLAB (version R2008a, The MathWorks, Natick, MA, USA), using a program written specifically for this purpose. From each scan, the maximum CMAP (amplitude and area of the negative peak), excitability and step variables were determined. To quantify excitability, we first determined the stimulus intensities that elicited the responses closest to 5%, 50%, and 95% of the maximum CMAP amplitude (S5, S50, and S95, respectively; see Figure 1A). From these values, we derived the absolute stimulus intensity range as the difference between S95 and S5, and the relative stimulus intensity range (absolute range divided by S5). We used S5 and S95 rather than S0 and S100 to minimize influence of noise (at the low end of the scan) and decrement effects (at the high end, see Discussion) on the excitability variables. Steps were quantified by the maximum step size (absolute size of the largest step in the scan, in μV, and relative size as percentage of the maximum CMAP amplitude), total step size (cumulative absolute size of the steps), step percentage (cumulative step size as a percentage of the maximum CMAP amplitude) and step number.

The maximum CMAP, S5, S50, S95 and the derived excitability variables were determined automatically by the program. The analysis of the steps was semi-automatic, in the sense that steps were identified by selecting them in the CMAP scans with a mouse click, after which the program automatically determined the values for the various step variables indicated above. For the purpose of identification and selection, steps were defined as clear gaps in the CMAP scan that were bounded by two plateaus (at the upper and lower end of the gap), each of which consisted of at least 3 consecutive responses of about the same size (i.e., disregarding noise). Steps smaller than 2% of the maximum CMAP and localized in the lower (<5%) or upper (>95%) end of the CMAP scan are probably caused by the activation of the first or last MUs with clearly distinct thresholds. These steps occur frequently in normal subjects as well as patients, and were, therefore, not included in the step variables as described above.

### Statistical analysis

SPSS (SPSS Inc, Chicago, IL, USA; version 15.0.1) was used for statistical analysis of the data. Because the data were not normally distributed, non-parametric paired tests (Friedman test and Wilcoxon signed ranks test) were used to assess significant differences in the CMAP scan variables and VAS scores between the different stimulus parameter settings (stimulus frequency, duration, and number). Since we performed multiple tests we used a significance level of *p* < 0.01.

## Results

In total, 98 CMAP scans were recorded. The effect of stimulus duration was not studied in 1 subject because of lack of time. All CMAP scans were of good quality for analysis. The median VAS score for CMAPs scans recorded with the common protocol (2 Hz, 0.1 ms and 500 stimuli) was 1.0 (range, 0–1.8) on a scale from 0 to 10. CMAP scans and derived variables were similar for the median and ulnar nerves, although there was a trend toward higher ulnar nerve excitability values (Table 1). There were no significant differences in the CMAP scan variables between Brisbane and Rotterdam, although step percentage (*p* = 0.03) tended to be lower in Brisbane (Table 1).

**Table 1:** Results (median and range) with standard protocol

**Variable**	**Rotterdam**	**Brisbane median**	**Brisbane ulnar**
	**(n = 11)**	**(n = 6)**	**(n = 6)**
**Max CMAP (mV)**	9.5 (6.9–13.5)	11.8 (6.5–12.6)	11.0 (9.7–14.4)
**S5 (mA)**	8.5 (6.4–16.0)	9.0 (7.8–13.9)	11.3 (8.7–22.1)
**S50 (mA)**	10.6 (8.2–20.9)	11.5 (9.6–16.0)	13.8 (11.6–27.1)
**S95 (mA)**	13.3 (9.6–24.5)	14.3 (11.2–20.3)	17.4 (15.6–33.6)
**Range (mA)**	5.0 (2.7–8.9)	5.3 (2.8–8.2)	7.6 (5.4–11.5)
**Relative Range**	0.44 (0.35–0.88)	0.53 (0.34–0.79)	0.63 (0.45–0.99)
**Step Number**	2 (0–7)	0.5 (0–5)	0.5 (0–4)
**Step percentage (% )**	4.9 (0.0–15.9)	0.3 (0–10.9)	0.3 (0–9.2)

Differences between centers and nerves were statistically not significant (*p* > 0.01).

We found no effect of stimulus frequency on the CMAP scan variables (*p* > 0.1) and VAS scores (*p* = 0.34) (Table 2). Stimulus duration affected S5, S50, S95, and the absolute and relative range significantly (*p* < 0.005), with higher stimulus intensity values for shorter stimulus durations (Table 3), but there was no relation with the step variables, maximum CMAP, and VAS score.

**Table 2:** Results (median and range) for different stimulus frequencies

**Variable**	**1 Hz**	**2 Hz**	**3 Hz**
	**(n = 11)**	**(n = 11)**	**(n = 11)**
**Max CMAP (mV)**	9.4 (6.9–13.2)	9.5 (6.9–13.5)	9.5 (6.9–12.5)
**S5 (mA)**	8.9 (5.9–22.0)	8.5 (6.4–16.0)	9.0 (6.0–18.6)
**S50 (mA)**	11.2 (8.5–29.0)	10.6 (8.2–20.9)	11.1 (8.3–26.6)
**S95 (mA)**	13.6 (9.7–34.3)	13.3 (9.61–24.5)	13.5 (9.5–31.1)
**Range (mA)**	5.0 (2.4–12.3)	5.0 (2.7–8.9)	4.5( 2.3–12.6)
**Relative Range**	0.50 (0.32–0.99)	0.44 (0.35–0.88)	0.49 (0.33–0.97)
**Step Number**	2 (0–5)	2 (0–7)	1 (0–6)
**Step percentage (% )**	4.7 (0–15.8)	4.9 (0–15.9)	4.0 (0–11.7)
**VAS**	1.0 (0.1–5.1)	1.0 (0.0–1.8)	1.2 (0.1–3.8)

Standard protocol was 500 stimuli, 2hz, 0.1 ms

Results for the different stimulus frequencies (1 Hz, 2 Hz, and 3 Hz). None of the differences between the parameters were significant.

**Table 3:** Results (median and range) for different stimulus durations

**Variable**	**0.05 ms**	**0.1**	**0.3 ms**
	**(n = 10)**	**(n = 11)**	**(n = 10)**
**Max CMAP (mV)**	10.9 (4.7–12.9)	9.5 (6.9–13.5)	10.9 (4.7–12.9)
**S5 (mA)**	13.4* (9.4–29.5)	8.5* (6.4–16.0)	4.4* (2.8–10.0)
**S50 (mA)**	16.1* (11.5–34.7)	10.6* (8.2–20.9)	5.2* (3.5–12.4)
**S95 (mA)**	18.8* (14.2–39.6)	13.3* (9.61–24.5)	6.3* (4.6–14.9)
**Range (mA)**	5.6* (3.8–10.1)	5.0* (2.7–8.9)	2.0* (1.6–4.8)
**Relative Range**	0.46* (0.27–0.61)	0.44* (0.35–0.88)	0.51* (0.34–0.70)
**Step Number**	2 (0.8)	2 (0–7)	1.5 (0–5)
**Step percentage (% )**	1.9 (0–5.5)	4.9 (0–15.9)	2.9 (0–10)
**VAS**	1.2 (0.0–3.2)	1.0 (0.0–1.8)	1.0 (2.7–2.4)

The excitability parameters all differed significantly (*p* < 0.01) between all stimulus durations settings (indicated with *). For example, S5 with 0.05 ms differs significantly from 0.1 ms and from 0.3 ms.

Table 4 shows the results for the step variables as a function of the number of stimuli. The 500-stimuli and 1000-stimuli CMAP scans proved to be similar. Differences in step percentage existed primarily between these and the 300-stimuli scans (*p* = 0.005 for 300 vs 500 stimuli and *p* = 0.004 for 300 vs 1000 stimuli). Differences in step number were not significant at the *p* = 0.01 level, but showed a clear trend towards increasing step number with decreasing stimulus number. The excitability variables did not change with stimulus number.

**Table 4:** Results (median and range) for different stimulus numbers

**Variable**	**300**	**500**	**1000**
	**(n = 12)**	**(n = 12)**	**(n = 12)**
**Max CMAP (mV)**	11.7 (6.5–13.8)	11.6 (6.5–14.4)	11.4 (6.6–14.2)
**S5 (mA)**	9.6 (8.4–22.9)	10.0 (7.8–22.1)	9.9 (7.8–23.3)
**S50 (mA)**	11.9 (10.0–28.8)	12.4 (9.6–27.1)	12.2 (10.1–29.6)
**S95 (mA)**	16.3 (11.0–35.1)	17.2 (11.2–33.6)	16.8 (11.3–35.8)
**Range (mA)**	5.8 (2.3–12.15)	6.2 (2.8–11.5)	6.4 (2.8–12.5)
**Relative Range**	0.59 (0.27–0.95)	0.61 (0.34–0.99)	0.62 (0.33–0.89)
**Step Number**	1.5 (0–7)	0.5 (0–5)	0.0 (0–3)
**Step percentage (% )**	3.9 (0.0–16.5)	0.3* (0.0–10.9)	0.0* (0.0–10.9)

The ulnar nerve was stimulated in 6 patients and in the other 6 patients the median nerve was stimulated. Significant (*p* < 0.01) differences with a stimulus number of 300 are indicated with an *. Other differences between the different stimulus numbers were not significant.

## Discussion

This study examined the stimulus parameters involved in the CMAP scan, aiming to optimize its quantification and minimize subject discomfort. Our results show that both stimulus duration and number of stimuli need to be standardized in CMAP scan studies, to ensure that data of different studies can be compared and normative data established. As is already known, stimulus duration has an effect on the excitability variables (Table 3) [4-6]. For the CMAP scan, determination of an optimal value for this parameter is somewhat arbitrary, however. A shorter stimulus duration decreases the steepness of the CMAP scan (the slope of the steep portion of the scan, expressed in mV/mA) and MU thresholds become more separated. Furthermore, the resolution of our stimulator is limited to 0.01 mA; smaller increases in stimulus intensity cannot be set. This implies that, if the absolute range of a CMAP scan is less than 5 mA, some intensities will be used more than once for stimulation with 500 stimuli. Even at stimulus duration of 0.1 ms, this is quite common. In other words, using shorter stimulus durations will allow sampling at a greater number of different stimulus intensities and thereby increase the resolution of the CMAP scan. The disadvantage of using short stimulus durations such as 0.05 ms is that the machine-imposed safety limit of 100 mA is reached more easily. This implies that in patients with increased thresholds such as in Guillain-Barré syndrome, the high end of the CMAP scan may be missed. Based on these practical issues, we therefore recommend for the CMAP scan stimulating with a pulse duration of 0.1 ms.

Stimulus frequency has no effect on the CMAP scan variables in healthy subjects (Table 2). However, in general, a high frequency increases the chance of decrements [8-10]. Decrements are systematic decreases in the CMAP in response to series of stimuli of equal strength. These may be due to disorders of neuromuscular transmission, but small decrements (<10% of the maximum CMAP area) can also occur in healthy subjects and patients with ALS. Such decrements in CMAP area extend over the first 5 to 50 stimuli, and are possibly due to an increased muscle fiber conduction velocity or changes in the nerve or muscle membrane [9,10]. Given the repetitive nature of the stimulation used for the CMAP scan, small decrements might therefore be expected and indeed were observed in our study, particularly during the first 5 stimuli. This decrement effect, which complicates CMAP scan quantification and interpretation, appears to be smaller for 2 Hz than for 3 Hz [9,10]. This would favour low-frequency stimulation. However, compared to 2 Hz, stimulating with 1 Hz doubles the recording time and, hence, increases the risk of movement artefacts. Stimulating with 2 Hz appears the best compromise to minimize decrements and movement artefacts at reasonable recording time. In this context, it should also be noted that the size of the decrement decreased with fixation of the thumb to the side of the hand. This constriction of movement reduces muscle fiber shortening, and hence limits the change in muscle fiber conduction velocity [11,12]. Fixation is, therefore, highly recommended.

The presence of multiple steps is a hallmark of abnormal CMAP scans [1,2,13]. It is, therefore, particularly important to standardize CMAP scan stimulation settings that may influence the step parameters. Differences in step percentage between scans with 300 and 500 stimuli and between scans with 300 and 1000 stimuli were significant. Differences in step number were not significant, but showed a clear trend (Table 4). The increase in step percentage and step number with decreasing stimulus number most likely results from undersampling of the steep part of the scan and, hence, the introduction of spurious steps. Differences in step percentage and step number between 500 and 1000 stimuli CMAP scans were small and not significant (Table 4). This suggests that 500 stimuli provide sufficient detail in the steepest part of the scan. Moreover, recording a CMAP scan with 1000 stimuli extends the duration of the test, which increases the risks of movement artefacts and patient discomfort. Hence, we prefer 500 stimuli for the recording of the CMAP scan. This does require, however, that the stimulus limits (S0 and S100) are set correctly so that most of the responses are in the steep portion of the scan and not in the tails. It should also be noted that specific applications, such as the Baysian MUNE analysis [3], may yet require 1000 or more stimuli.

During our previous work on the CMAP scan, we noted that a downwards recording direction appeared to ne better tolerated. Although downward recordings generally require exclusion of a number of decremental responses at the top of the CMAP scan from the analysis, at least this is feasible. In upward scanning, the effect of decrements cannot be separated from the variability in the scan that is due to alternation and MU recruitment. For that reason, and because it seems to be better tolerated, we consider downward recording preferable.

### Inter-center variability

There were no significant differences in the mean values of the CMAP scan variables between the two centers and their ranges were similar. Nevertheless, step variables showed a trend towards lower values in Brisbane. All data were analysed by the same researcher, so no interobserver variability can have occurred in the step detection. Differences could be the result of the small number of subjects in whom the median nerve was assessed in Brisbane (n = 6). Another factor that may explain the lower step values in Brisbane is that those CMAP scans had fewer responses at subthreshold stimulus intensities (the lower tail). This resulted in more stimuli in the middle part of the CMAP scan and, hence, fewer spurious steps and smaller step size.

## Conclusions

The optimal recording parameters for the CMAP scan are: stimulus duration of 0.1 ms, stimulus frequency of 2 Hz, and a stimulus number of 500 with a downwards recording direction. These stimulation settings optimize CMAP scan quantification and minimize subject discomfort, artefacts and recording time. Stimulus number and duration need to be standardized in future CMAP scan studies.

## Abbreviations

CMAP, Compound Muscle Action Potential; MU, Motor Unit; MUNE, Motor Unit Number Estimation; S0, Lowest stimulus intensity that elicits an all-or-none response of the lowest-threshold MU; S5, Stimulus intensitiy that elicits the response closest to 5% of the maximum CMAP amplitude; S50, Stimulus intensitiy that elicits the response closest to 50% of the maximum CMAP amplitude; S95, Stimulus intensitiy that elicits the response closest to 95% of the maximum CMAP amplitude; S100, Lowest stimulus intensity at which the maximum CMAP amplitude is recorded; VAS, Visual Analogue Scale.

## Competing interest

The authors have no competing interests to declare.

## Authors’ contributions

EMM carried out the measurements in Rotterdam, performed the statistical analysis and wrote the manuscript. RDH participated in the design of the study, carried out and coordinated the measurements in Brisbane, and revised the manuscript. JD assisted with the data acquisition, interpreting the results and writing and revising the manuscript. NMH carried out measurements in Brisbane and revised the manuscript. JRD participated in the design of the study and revising of the manuscript. JHB participated in the design of the study and writing and revising of the manuscript. GHV participated in the design of the study, statistical analysis and revising of the manuscript. All authors read and approved the final manuscript.
